# Weight outcomes of NU-HOME: a randomized controlled trial to prevent obesity among rural children

**DOI:** 10.1186/s12966-022-01260-w

**Published:** 2022-03-19

**Authors:** Jayne A. Fulkerson, Melissa Horning, Daheia J. Barr-Anderson, Abbey Sidebottom, Jennifer A. Linde, Rebecca Lindberg, Sarah Friend, Jennifer Beaudette, Colleen Flattum, Rebecca L. Freese

**Affiliations:** 1 School of Nursing, University of Minnesota, 5-140 Weaver-Densford Hall, 308 Harvard Street SE, Minneapolis, MN 55455 USA; 2 School of Kinesiology, University of Minnesota, Minneapolis, MN USA; 3 Care Delivery Research, Allina Health, Minneapolis, MN USA; 4 Division of Epidemiology and Community Health, School of Public Health, University of Minnesota, Minneapolis, MN USA; 5 Minneapolis Heart Institute Foundation, Minneapolis, MN USA; 6 Biostatistical Design and Analysis Center, Clinical and Translational Science Institute, University of Minnesota, Minneapolis, MN USA

**Keywords:** Family meals, Prevention, Childhood obesity, Healthy eating, Home food environment, Physical activity, Body fat, BMI, Community, Rural

## Abstract

**Background:**

Rural children are at greater obesity risk than their urban peers. The NU-HOME study is an innovative collaborative effort to prevent childhood obesity in rural communities. Weight outcomes of the NU-HOME study, a family-meal focused randomized controlled trial (RCT) are described. We hypothesized that compared to control group children, intervention group children would have significantly lower weight-related post-intervention (PI) outcomes.

**Methods:**

Participants were 114 dyads (7–10 year-old rural children and a parent). In 2017–2018 and 2018–2019, research staff measured height, weight and body fat at baseline (BL) and PI. Families were randomized to intervention (*n* = 58) or control (*n* = 56) groups without blinding. Designed with Social Cognitive Theory and community engagement, the NU-HOME program included seven monthly sessions delivered in community settings and four goal-setting calls. The program engaged entire families to improve healthy eating, physical activity, family meals and the home food environment. Multiple linear and logistic regression models tested PI outcomes of child BMIz-score, percent body fat, percent over 50th percentile BMI, and overweight/obesity status by treatment group, adjusted for BL values and demographics (*n* = 102).

**Results:**

No statistically significant intervention effects were seen for child BMIz or overweight/obesity status. However, a promising reduction in boys’ percent body fat (− 2.1, 95% CI [− 4.84, 0.63]) was associated with the intervention.

**Conclusions:**

Although our findings were in the hypothesized direction, making significant impacts on weight-related outcomes remains challenging in community trials. Comprehensive family-focused programming may require intensive multi-pronged interventions to mitigate complex factors associated with excess weight gain.

**Clinical trial registration:**

This study is registered with NIH ClinicalTrials.gov: NCT02973815.

**Supplementary Information:**

The online version contains supplementary material available at 10.1186/s12966-022-01260-w.

## Background

Rural community populations (i.e., “any population not in an urban area” that is defined by a population of less than 2500) [1] in the United States (U.S.) have higher health risks associated with cardiovascular disease/mortality [2] and obesity [3] than urban and suburban populations [4, 5]. In fact, national obesity rates are higher for both rural youth and adults [6, 7] compared to their urban counterparts. Similar findings are demonstrated at the state and county level. For example, in Minnesota, U.S., obesity prevalence is higher in nonmetro or rural counties compared to urban counties [8]. Specific behavioral risk factors tied to these disparities include poor dietary intake [5, 9] and low physical activity levels [2, 3, 5, 9].

Research has shown that some of the rural/urban health differences may stem from less availability and access to healthy and affordable foods [10, 11], lack of nutrition education and weight management services [12, 13], and fewer opportunities and access to facilities to be physically active [10, 11] with fewer activity resources and a strong reliance on automobiles to meet transportation needs. However, rural communities have many assets as well, including close ties and supportive networks of caring individuals [14, 15]. Many of these communities also have a strong infrastructure for volunteerism [14, 15].

To date, there is a very limited number of randomized controlled trials (RCTs) devoted to preventing childhood obesity in rural communities by engaging children and parents together. An 11-week, two group, randomized obesity prevention pilot study of 6–9-year-old youth on a rural American Indian reservation intervened with children on physical activity, nutrition, sleep and screen time approximately three times per week and saw promising effects on body mass index (BMI) z-scores. Parent engagement was relatively limited and occurred through an informational meeting (1 h, once during the study), take-home resource toolkits (3 times per week as parents picked up their children from sessions), text messages (once per week), with monthly family dining and activity events. Relatively high percentages of families participated in the family nights (100, 80, 80% for first, second and third events, respectively), one-third of families participated in home activities, slightly over half participated in study information sessions [16]. The E-FLIP for Kids randomized controlled trial aimed to address child weight, dietary intake, glycated hemoglobin and quality of life for 8–12-year-old children through a program for children with overweight or obesity and their parents that included 20 group contacts over a year. Study findings showed no significant differences in BMI z-scores between treatment groups over a two year period [17]. Increased BMI z-scores were seen for the intervention group compared to the control group in the iCook 4-H RCT that consisted of 9–10-year-old child and adult dyads and promoted cooking, eating and playing together in six 2-h biweekly sessions [18]. This limited evidence base suggests more work is needed to engage parents and address childhood obesity in rural communities.

The present study describes program effectiveness of the NU-HOME RCT from baseline to post-intervention. The NU-HOME intervention program was developed to prevent obesity in 7–10-year-olds, engaged whole families, was delivered in community settings, and had a primary outcome of BMI z-scores. We hypothesized that compared to children in the wait-list control group, children in the intervention group would have significantly lower weight-related post-program outcomes (BMI z-score, body fat percentage, incidence of overweight/obesity).

## Methods

### Trial design

The New Ulm at HOME (NU-HOME) study was designed as a 5-year, two-group RCT to reduce excess weight gain among 7–10-year-old children living in rural communities by promoting family meals, healthy home food environments and healthy eating and physical activity as a family. A staggered-cohort design was used with two cohorts recruited one year apart (2017 and 2018) [19].

The NU-HOME study involved a collaborative effort between researchers at the University of Minnesota (UMN), Allina Health, the Minneapolis Heart Institute Foundation (MHIF) and community partners. The UMN research team had conducted RCTs to prevent childhood obesity in urban communities [20–23]. Allina Health and MHIF had conducted a 10-year community demonstration project called Hearts Beat Back: Heart of New Ulm Project (HONU) to reduce prevalence of adult myocardial infarctions and cardiovascular risk factors in a rural community [24, 25]. The NU-HOME study collaboration included using the academic childhood obesity research infrastructure while leveraging existing stakeholder groups and research and community infrastructure of HONU. In 2016, the NU-HOME research team created a study-related Action Team by building on the established HONU leadership group that included local health systems, schools, public health departments and other stakeholders. The research team and Action Team worked together for a year to complete program adaptation, including content revision, and logistical planning that included identifying key strategies for recruitment and implementation [26]. The full scale RCT followed this planning and adaptation year. These collaborations enhanced the research team’s credibility and established community trust. Additional details are published elsewhere [19]. Together, these experiences and resources provided an opportunity to create a childhood obesity program relevant to rural community needs, recruit participants and conduct data collection effectively, and ensure the program could be sustained in the community after the research was completed [27].

### Participants

Our comprehensive recruitment strategy included distribution of flyers at pediatric clinics and community sites, study information posted in community education brochures, informational sessions at community events, and letters mailed to families with children in the eligible age range served by the local health system and signed by a pediatrician (who was also a member of the Action Team). Study promotion also occurred through marketing channels, distribution through children’s backpacks from school, local newspapers and other communications formats.

Eligible NU-HOME study participants included 7–10-year-old children and a parent/guardian (hereafter referred to as parents) who lived within a 50-mile radius of the rural New Ulm or Sleepy Eye, Minnesota communities. In our previous urban RCT with 8–12-year-old children, we showed significant BMI z-score change in prepubertal children [22]. Thus, for the NU-HOME trial, we recruited 7–10 year old children to increase the chance of having a prepubertal sample. Inclusion criteria included willingness to be randomized to an intervention or wait-list control group; willingness to attend data collection visits; willingness to attend seven monthly intervention sessions if randomized to the intervention group; and requirement that the parent live with the child at least half-time and prepare most of the family’s meals. Exclusion criteria were planning on moving from the area in the next 6 months and the existence of a medical condition(s) or food allergies contraindicating intervention program participation. Staff and volunteers recruited 114 child/parent dyads across the two cohorts. Participants in cohort one (*n* = 60 dyads) and cohort two (*n* = 54 dyads) were from the city of New Ulm, Minnesota and 11 surrounding communities. The analytic sample for the NU-HOME main trial outcomes evaluation includes the 102 children (89% of the sample) with BMI and 101 children with body fat data post program.

### Procedures and measures

Interested participants were called by research staff for eligibility screening and to schedule a visit for formal enrollment and baseline data collection. Consent and assent forms and a validated Home Food Inventory (HFI) [28] were mailed to parents in advance of the in-person visit held in the local public schools. Parents completed the HFI at home prior to the data collection visit and provided it to the research team after completing the consent and enrollment process at the data collection visit. Parent and child participants provided written consent/assent for study participation. Post-intervention data collection occurred 8 to 10 months after baseline.

Trained and certified research staff measured child and parent height using a stadiometer, and weight and body fat using Tanita scales (TBF400-Total Body Composition Analyzer), with standardized procedures [29]. BMI [weight (kg)/height (m^2^)], age- and sex-adjusted BMI percentiles, and standardized z-scores (ANTHRO 1.02 software-CDC) were calculated [30]. Percent over 50th percentile BMI was calculated by finding the percentage of each child’s BMI above the age- and sex-matched BMI of a child at the 50th percentile [31, 32], based on the CDC BMI percentiles [33]. Child weight status categories were created based on CDC BMI percentiles for age and sex (Underweight = less than 5th percentile, Normal or Healthy weight = 5th percentile to less than 85th percentile, Overweight = 85th percentile to less than 95th percentile, Obese = 95th percentile or greater). Parent weight status categories were based on BMI (Underweight = BMI less than 18.5, Normal or Healthy weight = BMI 18.5 to less than 25, Overweight = BMI 25 to less than 30; Obese = BMI 30 or more).

A parent survey included validated food and activity measures, questions about parent’s perceptions and demographic characteristics [19]. Parent report of demographic characteristics relevant to this report include sex and race/ethnicity of participating children and parents; child pubertal status; parent education and marital status; and household economic assistance (a socioeconomic proxy for economic wealth reported as either receiving public assistance or free/reduced lunch) and food insecurity. Food insecurity was measured at the household level with the six-item food security survey from the UDSA. Questions asked about the past 12 months and included items about whether the food families bought did not last and if they could afford to eat balanced meals as well as items that asked about changes they may have made because there was not enough money for food such as eating less or skipping meals [34, 35].

Other data collection activities and procedures, including child 24-h dietary recalls, child accelerometry, child psychosocial surveys, and real-time data collection of frequency and quality of family meals and snacks are not reported here and are described in detail elsewhere [19]. We used University-supported Research Electronic Data Capture (REDCap) software (http://www.project-redcap.org/) and data were saved directly to UMN password-protected servers. Data collection visits took 1.5–2 h. Families received a $25 gift card for each in-person data collection visit, plus additional gift cards for completing other measures for a total of up to $75 for each measurement visit [19]. All recruitment and data collection procedures and materials were approved by the UMN Institutional Review Board. All intervention related activities were approved by Quorum Institutional Review Board (an external IRB for Allina Health).

### Randomization

**Fig. 1:**
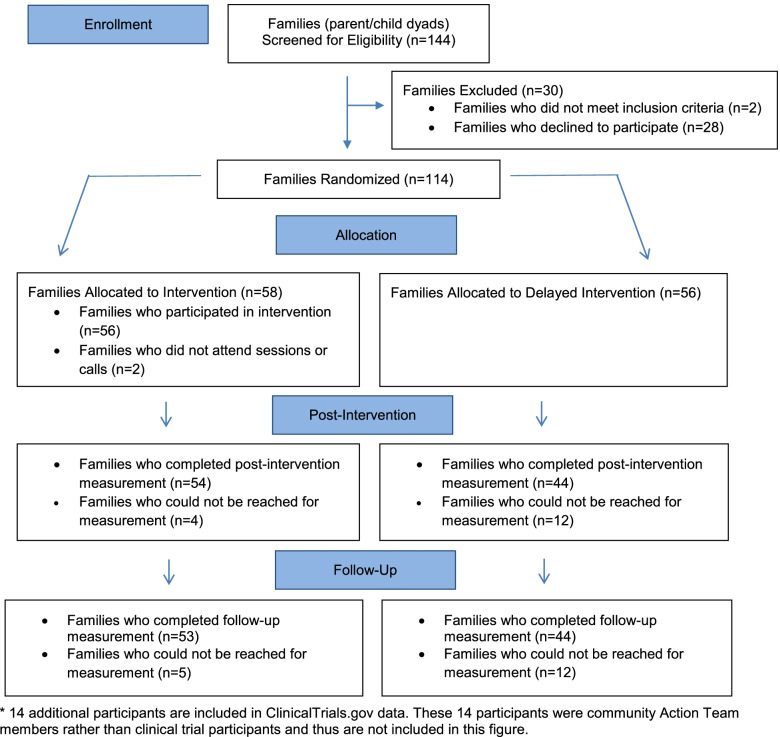
NU-HOME Consort Flow Diagram for Randomized Clinical Trial. This figure was published in Contemporary Clinical Trials, Vol 100, Fulkerson JA, Horning ML, Barr-Anderson DJ, Linde JA, Sidebottom AC, Lindberg R, Friend S, Flattum C, Freese RL. Universal childhood obesity prevention in a rural community: Study design, methods and baseline participant characteristics of the NU-HOME randomized controlled trial, Copyright Elsevier (2021)

After baseline data collection, families were randomized to either the NU-HOME program (*n* = 58 child/parent dyads) or the wait-list control (*n* = 56 child/parent dyads) by the study statistician using a computer-generated randomization schedule in Stata version 15 (StataCorp LLC, College Station, TX). The research team was not blinded to study group assignment owing to the nature of the behavioral intervention. Families randomized to the wait-list control group were invited to participate in an abbreviated program after all data collection was complete for their cohort. See Fig. 1 for recruitment and retention information.

### Intervention program

We adapted our team’s previous HOME Plus intervention program shown to be effective in reducing excess weight gain among prepubertal youth [22]. The urban HOME Plus study was a two-arm randomized controlled trial that included 10 monthly two-hour sessions and five goal-setting telephone calls to promote family meals and healthy eating to prevent childhood obesity [36]. The program engaged whole families and was delivered in community settings. For the NU-HOME trial in rural communities, adaptations were made to the program, goals and behavioral messages [36] using Social Cognitive Theory (SCT) [37], a socio-ecological framework [38, 39], and community engagement [40] to address challenges that rural families might encounter related to eating healthy foods and family meals. The scientific premise was that in addition to including healthful eating and physical activity, identified as key intervention components in reviews published at that time [30, 41, 42], modifiable behaviors under parents’ control such as types of foods served and accessible at home and meal preparation were logical targets for obesity prevention. Intervention session activities were designed to enhance child and parent skills, self-efficacy, and outcome expectations related to trying new vegetables, cooking healthful meals, sharing mealtimes, and participating in physical activities together.

Our NU-HOME program had three primary goals: 1) eating more healthy family meals, eating more fruits and vegetables at meals, and eating proper portion sizes; 2) increasing the healthfulness of food available at home (more fruits and vegetables, fewer sugar-sweetened beverages available, and fewer high fat and high sugar foods in the home); and 3) engaging in positive activities as a family, including physical activity, having positive conversations at family meals, turning off the TV and not using phones or tablets or computers during family meals, and cooking together. Although these goals are associated with specific outcomes and will be reported in future manuscripts, the focus of the present study was on the main outcome of child BMI z-score (see outcomes section below). The program was designed with seven, 2-h, monthly in-person sessions (October–April) with multiple family groups in community settings and focused heavily on experiential learning and skill building. Each session was taught multiple times each month to accommodate family schedules and about six families attended each session. These sessions included taste testing of vegetables, a family physical activity, family goal setting, separate parent and child sessions focused on activities around the monthly topic and ended with eating a family dinner together. The intervention also included four phone calls conducted individually with parents (after sessions 1, 3, 5 and 7) using motivational interviewing techniques; parents selected one or more of the goals described above [43]. More information about the intervention program can be found elsewhere [19, 36].

Assessment of process variables such as program fidelity, treatment dose and satisfaction are important to ensure interventions are delivered as intended and that participant attendance and satisfaction are as expected [44, 45]. Fidelity assessments comprised of intervention staff checklists noting completion of session activities as well as trained non-intervention staff observations of session activities were completed. Session and parent phone call attendance were recorded in REDCap by intervention staff. On the process evaluation survey administered to intervention parents, parents were asked whether they would recommend the NU-HOME intervention study to other families with response options of yes or no. Parents were also asked to rate their satisfaction with the NU-HOME program on a 4-point Likert scale with response options of very dissatisfied, dissatisfied, satisfied and very satisfied.

### Outcomes

The primary outcome measures were age- and sex-adjusted child BMI z-score and body fat percentage at post-intervention. The childhood obesity treatment literature also often reports changes in percent over 50th percentile BMI for samples with negatively skewed BMI distributions [31, 32, 46]. Thus, given the higher prevalence of overweight and obesity in rural communities, we assessed this as a secondary outcome. Incidence (new cases) of overweight and progression of obesity to more severe levels are also important [47] and was also evaluated as a secondary outcome.

### Sample size/power

Sample size and power were calculated for the primary study outcome of age- and sex-adjusted child BMI z-score. Power calculations were based on two assessment time points, correlation over time, and variability of age- and sex-adjusted BMI z-scores. The recruitment of 114 families allowed for a 13% attrition rate for the final effective sample of about 100 families. Within-child correlation (ρ) of outcome measures over time were found to be 0.90 in our previous HOME Plus trial. Using a baseline-adjusted analysis approach will allow detection of an effect size of 0.25 for age-and sex-adjusted BMI z-score between groups with a sample size of 96 (48 per group) with 80% power at the 0.05 significance level. This effect size corresponds to a 1.4 kg decrease in average weight gain between intervention and control groups.

### Statistical analysis

Multiple linear regression models with an intent-to-treat analytic approach were constructed for the child weight-related outcomes of post-intervention BMIz- score, percent body fat, and percent over 50th percentile BMI, with the predictor of interest being the randomization group. All models were adjusted for the outcome measure at baseline, child sex, child age at baseline, and baseline household economic assistance. Incidence of increasing weight category was evaluated by defining a binary variable indicating whether or not a child increased any weight categories from baseline to post-intervention. Logistic regression was used with this binary variable as the outcome, adjusting for baseline overweight or obese weight status, randomization group, baseline child age and sex, and family economic assistance. These models used post-intervention data with the exception of four cases where the last observation was carried forward from a mid-intervention visit (within 5–6 months of post-intervention data collection) because these participants were missing post-intervention outcome data. Additionally, the “dose” or minutes of contact with each family was made into a high vs low categorical variable based on a natural split in a histogram among those in the intervention group. We secondarily assessed whether or not the dose category had any effect on post-intervention BMIz-score, adjusted for baseline BMIz-score, child sex, baseline child age, and baseline household economic assistance. Lastly, for the sake of interpretability, unadjusted change in weight (kg) was compared between the randomization groups using a t-test. All *p*-values are two-sided and considered at the 0.05 level for statistical significance. Analyses were carried out in R, version 3.6.1. (R Core Team (2019) [48].

## Results

### Sample characteristics

Table 1 presents the analytic sample characteristics as well as characteristics by treatment group. Adult participants primarily identified as female, with an average age of about 38 years, most were white and approximately three-quarters had overweight or obesity. Child participants were 58.8% girls, about 9 years old on average, primarily white and slightly less than half had overweight or obesity. Level of food insecurity was about one in five families, with more receiving some form of economic assistance.

**Table 1:** NU-HOME study baseline child, parent and household characteristics for the analytic sample (complete cases for the BMI z-score model) and by treatment group

CHILD Characteristics	Total Analytic Sample (*n* = 102)^a^	Intervention Group (*n* = 56)	Control Group (*n* = 46)
**Age (years)**			
Mean (SD)	9.0 (1.1)	9.0 (1.0)	9.0 (1.1)
Median [Range]	8.9 [6.9, 11.0]	8.8 [6.9, 11.0]	8.9 [7.0, 11.0]
**Sex, N (%)**			
Girls	60 (58.8)	30 (53.6)	30 (65.2)
Boys	42 (41.2)	26 (46.4)	16 (34.8)
**Ethnicity, N (%)**			
Not Hispanic/Latino	94 (92.2)	52 (92.9)	42 (91.3)
Hispanic/Latino	8 (7.8)	4 (7.1)	4 (8.7)
**Race, N (%)**			
BIPOC	7 (6.9)	5 (8.9)	2 (4.3)
White	95 (93.1)	51 (91.1)	44 (95.7)
**BMI Category, N (%)**			
Normal or Healthy weight	56 (54.9)	33 (58.9)	23 (50.0)
Overweight	22 (21.6)	11 (19.6)	11 (23.9)
Obese	24 (23.5)	12 (21.4)	12 (26.1)
**BMI z-score**			
Mean (SD)	0.8 (1.0)	0.7 (1.0)	1.0 (0.9)
Median [Range]	0.9 [−1.4, 3.0]	0.5 [−1.4, 3.0]	1.0 [−0.9, 2.6]
**% Over 50% BMI**			
Mean (SD)	19.3 (24.6)	16.7 (24.8)	22.3 (24.2)
Median [Range]	11.9 [−14.9, 107.8]	7.2 [−14.9, 107.8]	15.6 [−8.6, 87.8]
**% Body Fat**			
Mean (SD)	23.0 (7.6)	22.3 (7.6)	23.8 (7.5)
Median [Range]	20.8 [13.5, 46.6]	20.4 [13.5, 46.6]	21.7 [14.9, 45.0]
**Puberty, N (%)**			
Not started puberty	90 (88.2)	52 (92.9)	38 (82.6)
Started puberty	1 (1.0)	0 (0.0)	1 (2.2)
Unsure	11 (10.8)	4 (7.1)	7 (15.2)
**PARENT/HOUSEHOLD Characteristics**			
**Age (years)**			
Mean (SD)	38.0 (5.4)	37.9 (5.1)	38.1 (5.8)
Median [Range]	38.1 [27.3, 55.0]	38.0 [28.2, 51.0]	38.3 [27.3, 55.0]
**Sex, N (%)**			
Women	99 (97.1)	55 (98.2)	44 (95.7)
Men	3 (2.9)	1 (1.8)	2 (4.3)
**Ethnicity, N (%)**			
Not Hispanic/Latino	97 (95.1)	54 (96.4)	43 (93.5)
Hispanic/Latino	5 (4.9)	2 (3.6)	3 (6.5)
**Race, N (%)**			
BIPOC	2 (2.9)	1 (1.8)	2 (4.3)
White	99 (97.1)	55 (98.2)	44 (95.7)
**Marital Status, N (%)**			
Married or not married living with significant other	87 (85.3)	45 (81.4)	42 (91.3)
Separated, divorced, widowed, never married	15 (14.7)	11 (19.6)	4 (8.7)
**Education, N (%)**			
Less than or equal to High School	11 (10.9)	5 (9.1)	6 (13.0)
Some college	21 (20.8)	12 (21.8)	9 (19.6)
Associates degree	13 (12.9)	9 (16.4)	4 (8.7)
Bachelor’s degree or higher	56 (55.4)	29 (52.7)	27 (58.7)
Missing	1	1	0
**BMI Category, N (%)**			
Normal or Healthy weight	24 (24.0)	15 (27.8)	9 (19.6)
Overweight	36 (36.0)	22 (40.7)	14 (30.4)
Obese	40 (40.0)	17 (31.5)	23 (50.0)
Missing	2	2	0
**BMI**			
Mean (SD)	29.9 (6.9)	28.5 (5.9)	31.6 (7.6)
Median [Range]	28.6 [19.6, 51.6]	27.8 [19.6, 44.0]	30.0 [20.7, 51.6]
Missing (pregnant)	2	2	0
**% Body Fat**			
Mean (SD)	34.8 (8.8)	33.7 (8.6)	36.0 (9.0)
Median [Range]	33.7 [19.8, 53.2]	31.0 [20.8, 50.8]	36.2 [19.8, 53.2]
Missing (pregnant or had pacemaker)	3	3	0
**Food Insecurity **[34]**, N (%)**			
Insecure	19 (18.6)	11 (19.6)	8 (17.4)
Secure	83 (81.4)	45 (80.4)	38 (82.6)
**Economic Assistance, N (%)**			
Receives public assistance or free/reduced lunch	31 (30.4)	19 (33.9)	12 (26.1)
Does not receive public assistance or free/reduced lunch	71 (69.6)	37 (66.1)	34 (73.9)

*BIPOC* Black, Indigenous, People of Color

^a^One child did not have body fat data collected. Incidental missing data for parent information

### Program attendance and Fidelity

**Fig. 2:**
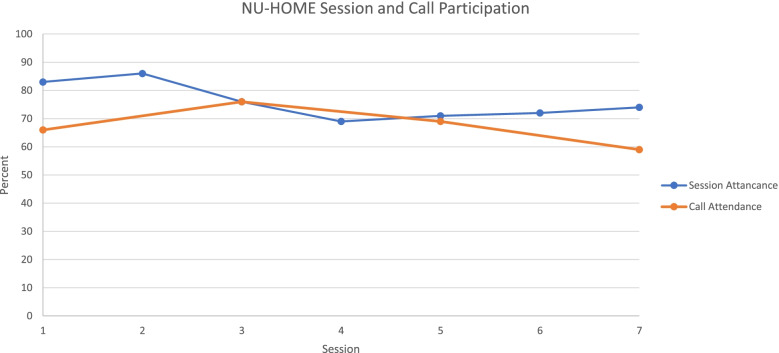
Percentage of Family’s Attending Monthly In-Person Intervention Sessions and Motivational Phone Calls (*n* = 58 families)

Figure 2 shows measures of treatment dosage, including family attendance at the in-person, monthly sessions from October to April which averaged 76%. Completion of motivational calls are also shown with average completion of 67%. We did not see a statistically significant effect of dose on post-intervention child BMIz-score among those in the intervention group.

Research team members assessed program fidelity at sessions 2, 4 and 6 and found high fidelity across sessions, indicating the program was implemented as intended [45, 49]. At post-intervention, parents in the intervention group (*n* = 52) reported a high level of satisfaction with the NU-HOME program; 73% (*n* = 38) reporting they were very satisfied, 27% (*n* = 14) were satisfied and 0% (*n* = 0) were dissatisfied or very dissatisfied. All parents (*n* = 52, 100%) in the intervention group reported they would recommend the NU-HOME study to a friend.

### Treatment group differences

Unadjusted change in weight (kg) showed a small, but statistically significant difference between groups (mean weight change: 4.31 kg increase control group, 3.16 kg increase intervention group, *p* = 0.031). Adjusted linear regression showed no significant differences between intervention and wait-list control groups in post-intervention child BMI z-score, body fat percentage or percent over 50th percentile BMI (Table 2). Similarly, there were no statistically significant group differences observed for incidence of increasing weight category (Table 2). However, differences were in the expected direction with intervention group participants having half the odds of increasing weight category (were at lower risk) compared to those in the wait-list control group. The outcome of body fat percentage showed the biggest difference between our intervention and wait-list control groups. Child sex was a statistically significant covariate in the overall model with body fat percentage as an outcome, therefore, we conducted analyses that stratified by sex to assess differences in body fat percent change by treatment groups for male and female participants separately. Stratified analyses indicate a larger program effect on boys in the intervention group compared to boys in the wait-list control group, although differences were not statistically significant (Table 3).

**Table 2:** Post-Intervention Linear and Logistic Regression Weight-Related Outcomes by Treatment Group (*n* = 102)

**Linear Regression Outcomes**	**Intervention Mean (SD)**	**Control Mean (SD)**	**Adjusted Difference**^**a**^	**95% Confidence Interval**	**P-value**
BMI z-score	0.69 (1.00)	1.03 (0.89)	−0.06	(−0.19, 0.07)	0.342
Body fat percentage	23.68 (8.56)	26.50 (8.86)	−1.37	(−3.17, 0.43)	0.135
Percent over 50% BMI	17.03 (25.37)	24.17 (25.67)	−1.61	(−4.29, 1.07)	0.235
**Logistic Regression Outcome**	**Intervention N (%)**	**Control N (%)**	**Estimated Odds Ratio**	**95% Confidence Interval**	**P-value**
Incidence of increasing weight status^b^	4 (7.14)	6 (13.04)	0.49	(0.11, 1.91)	0.311

All models adjusted for baseline value of outcome, child age, child sex, and family economic assistance

^a^Adjusted difference for the control group in reference to the intervention group

^b^Incidence of increasing weight status is defined as a binary variable and assessed whether or not a child increased in weight status categories from baseline to post-intervention (e.g., healthy weight to overweight or obese, overweight to obese)

**Table 3:** Post-Intervention Child Percent Body Fat Stratified by Sex by Treatment Group (*n* = 101)

Sex	Intervention Mean (SD)	Control Mean (SD)	Adjusted Difference^**a**^	95% Confidence Interval	***P*-value**
Girls (*n* = 60)	25.27 (7.50)	28.42 (8.87)	−0.91	(− 3.33, 1.49)	0.449
Boys (*n* = 41)	21.85 (9.46)	22.65 (7.74)	−2.11	(−4.84, 0.63)	0.128

All models adjusted for baseline value of outcome, child age, and family economic assistance

^a^Adjusted difference for the control group in reference to the intervention group

## Discussion

The NU-HOME study was a collaborative effort to adapt a previous intervention program with urban communities to be relevant for rural communities and test its effectiveness in modifying childhood obesity. Although the intervention program engaged whole families and addressed known contributors to obesity such as unhealthy eating (high-calorie, low-nutrient foods and beverages), unhealthy home food environments (availability of unhealth foods in the home), and encouraged family physical activity and frequent family meals, our trial did not show statistically different weight-related outcomes between children in the intervention and wait-list control groups. We did show promising effects for body fat percentage at post-intervention by treatment group for boys, with lower body fat percentage for boys in the intervention group compared to boys in the wait-list control group. Research is needed to understand the clinical importance of body fat changes during preadolescence and whether body fat and other measures of body mass are important outcomes to assess in obesity prevention research. Our study’s null findings on BMI z-scores of school-age children are similar to the few other existing family-focused RCTs in rural communities [17, 18]. These findings indicate that it is challenging to make significant impacts on BMI z-scores and weight status in school-age children living in rural community settings.

Because baseline levels of weight-related outcomes among school-age children are strong drivers of future weight status, intensive family-focused prevention programming may require extensive societal policy and environmental interventions to mitigate complex factors that lead to excess child weight gain. An overview of Cochrane reviews [50] concluded that school-age children may be more influenced by surrounding obesogenic environments than younger children. Thus, BMI z-score and other weight-related outcomes may not be impacted in school-age preteens unless we actively intervene on all of the environments that influence their behaviors such as school environments, friends and friend’s homes, relative’s homes as well as the community food and activity environments [51] and wider determinants of health that perhaps include the influences of genetic make-up [52]. Substantial efforts to positively promote healthy eating and activity outside of the home and align them with home-based activities may be needed. School-age children may also be less autonomous than adolescents and more reliant on their parents, but the role of friends and parents at this age and how to best engage them is yet to be determined [50].

Families in rural communities may encounter some barriers to healthy eating and activity levels that are similar to families living in urban communities such as competing family priorities and concerns for child safety [16, 17]. However, they may also encounter unique barriers such as long driving distances to healthy food outlets and physical activity environments [53]. Although we provided gift cards to participating families in our intervention program to offset transportation costs, we did not offset the costs associated with grocery shopping for healthy foods and travel associated with physical activity and sports participation which could have limited integration of healthy eating and physical activity from intervention sessions into daily family lifestyle behaviors.

Although the NU-HOME study partnered with community and followed the success of HONU programming in the rural communities involved in our study, future studies may need to be multicomponent, and address the dynamic interrelations among various individual, interpersonal, organizational, community and policy influences on overall health of children and their families [51]. The prevention of childhood obesity and maintenance of healthy weight by promoting healthful diets, activity and environments is critical before obesity develops [51].

The present study had several limitations. The NU-HOME study included a self-selected sample who volunteered to participate and may not be generalizable to rural families nationally. The sample was relatively small with 102 families in the analytic sample and was fairly homogeneous. Although planned contact hours (session attendance and motivational calls) were substantial for our obesity prevention program (14 h in-person, 2 h by phone), participants did not receive the 26 contact hours recommended for childhood obesity weight-loss treatment programs [54], and perhaps more intensity is needed even for universal prevention programs given the prevalence of childhood obesity. Therefore, future intervention studies should meet the 26 minimum contact hours and perhaps be more frequent than once per month. Study strengths included the rigorous RCT study design; the unique community partnerships; objective measurement of weight-related outcomes; and excellent retention, satisfaction and good program attendance at the offered sessions. In addition, the intervention program included in-person experiential activities with both parents and children and used recommended motivational techniques [43] in phone calls with parents to facilitate behavior change. Furthermore, our study aimed to prevent childhood obesity by using a universal approach (we did not use BMI as eligibility criteria). Yet, although primary prevention is recommended based on high-quality RCT reviews [55], including children that represent the full weight status range in the same intervention program may impact intervention effectiveness if risk factors differ substantially by weight status for additional weight gain as they grow.

To guide future obesity prevention research for elementary school age children living in rural communities, findings from RCT reviews [42, 55] and from studies conducted to date with families living in rural communities suggest that perhaps a combination of intensity and sustainability is warranted. The NU-HOME intervention provided substantial in-person hours with parents but sessions occurred monthly and thus may not have been of adequate intensity to impact child BMI z-scores. In contrast, the study by Brown and colleagues on a rural Indian reservation had activities three times per week but occurred over a short 11-week timeframe [16]. Intensive, sustained programming along with policy support and community environments conducive to healthy eating and activity are imperative. Specific next steps include identification of the most effective intervention components of existing intervention programs [42] and identification of the best combination of system support through schools, communities, and health care systems. In addition, evidence suggests that, for 6–12-year-old children, a larger focus on physical activity may be beneficial for obesity prevention [55].

## Conclusion

Although findings from the NU-HOME study were in the hypothesized direction, the lack of statistically significant effects of our RCT reinforce how difficult it is to make significant impacts on child weight-related outcomes in community trials. Rigorous multi-pronged interventions with intensive family-focused programming may be required to mitigate complex factors associated with excess weight gain among children.

## Supplementary Information

**Additional file 1.**

## Data Availability

The datasets used in the current study are available to other scientists from the corresponding author on reasonable request at the time the study is no longer actively funded to the investigative team.

## Abbreviations

NU-HOME: New Ulm at HOME
RCT: Randomized Controlled Trial
BL: Baseline
PI: Post-intervention
BMI: Body mass index
UMN: University of Minnesota
MHIF: Minneapolis Heart Institute Foundation
HONU: Hearts Beat Back: Heart of New Ulm Project
HFI: Home Food Inventory
IRB: Institutional Review Board

## References

[1] Defining rural population: Health Resources & Services Adminstration; 2021 [Available from: hrsa.gov/rural-health/about-us/definition/index.html].

[2] Cross SH, Mehra MR, Bhatt DL, Nasir K, O'Donnell CJ, Califf RM (2020). Rural-urban differences in cardiovascular mortality in the US, 1999-2017. JAMA..

[3] Joens-Matre RR, Welk GJ, Calabro MA, Russell DW, Nicklay E, Hensley LD (2008). Rural-urban differences in physical activity, physical fitness, and overweight prevalence of children. J Rural Health.

[4] Johnson JA, 3rd, Johnson AM. Urban-rural differences in childhood and adolescent obesity in the United States: a systematic review and meta-analysis. Child Obes (Print). 2015;11(3):233–41. 10.1089/chi.2014.0085 25928227

[5] Davis AM, Bennett KJ, Befort C, Nollen N (2011). Obesity and related health behaviors among urban and rural children in the United States: data from the National Health and Nutrition Examination Survey 2003-2004 and 2005-2006. J Pediatr Psychol.

[6] Hales CM, Fryar CD, Carroll MD, Freedman DS, Aoki Y, Ogden CL (2018). Differences in obesity prevalence by demographic characteristics and urbanization level among adults in the United States, 2013-2016. JAMA..

[7] Lundeen EA, Park S, Pan L, O'Toole T, Matthews K, Blanck HM (2018). Obesity prevalence among adults living in metropolitan and nonmetropolitan counties - United States, 2016. MMWR.

[8] CDC. CDC Diabetes County Data Indicators, 2006–2017: Center for Disease Control and Prevention; 2020 [Available from: https://www.cdc.gov/diabetes/data/index.html].

[9] Euler R, Jimenez EY, Sanders S, Kuhlemeier A, Van Horn ML, Cohen D (2019). Rural-urban differences in baseline dietary intake and physical activity levels of adolescents. Prev Chronic Dis.

[10] Yousefian A, Leighton , Fox K, Hartley D. Understanding the rural food environment--perspectives of low-income parents. Rural Remote Health. 2011;11(2):1631. 21513422

[11] Seguin R, Connor L, Nelson M, LaCroix A, Eldridge G (2014). Understanding barriers and facilitators to healthy eating and active living in rural communities. J Nutr Metab.

[12] Findholt NE, Davis MM, Michael YL (2013). Perceived barriers, resources, and training needs of rural primary care providers relevant to the management of childhood obesity. J RuralHealth.

[13] Shaikh U, Nettiksimmons J, Romano P (2011). Pediatric obesity management in rural clinics in California and the role of telehealth in distance education. J Rural Health.

[14] Meit M (2018). Exploring strategies to improve health and equity in rural communities.

[15] Fleming AR, Ysasi NA, Harley DA, Bishop ML. Resilience and Strengths of Rural Communities. 2018. In Harley D, Ysasi N, bishop M Fleming a (eds) disability and vocational rehabilitation in rural settings. Springer, Cham 10.1007/978-3-319-64786-9_7.

[16] Brown B, Harris KJ, Heil D, Tryon M, Cooksley A, Semmens E (2018). Feasibility and outcomes of an out-of-school and home-based obesity prevention pilot study for rural children on an American Indian reservation. Pilot and Feasibility Stud.

[17] Janicke DM, Lim CS, Perri MG, Mathews AE, Bobroff LB, Gurka MJ (2019). Featured article: behavior interventions addressing obesity in rural settings: the E-FLIP for kids trial. J Pediatr Psychol.

[18] White AA, Colby SE, Franzen-Castle L, Kattelmann KK, Olfert MD, Gould TA (2019). The iCook 4-H study: an intervention and dissemination test of a youth/adult out-of-school program. J Nutr Educ Behav.

[19] Fulkerson JA, Horning ML, Barr-Anderson DJ, Linde JA, Sidebottom AC, Lindberg R (2020). Universal childhood obesity prevention in a rural community: study design, methods and baseline participant characteristics of the NU-HOME randomized controlled trial. Contemp Clin Trials..

[20] Fulkerson JA, Rydell S, Kubik MY, Lytle L, Boutelle K, Story M (2010). Healthy home offerings via the mealtime environment (HOME): feasibility, acceptability, and outcomes of a pilot study. Obesity (Silver Spring).

[21] Fulkerson JA, Neumark-Sztainer D, Story M, Gurvich O, Kubik MY, Garwick A (2014). The healthy home offerings via the mealtime environment (HOME) plus study: design and methods. Contemp Clin Trials.

[22] Fulkerson JA, Friend S, Flattum C, Horning M, Draxten M, Neumark-Sztainer D, et al. Promoting healthful family meals to prevent obesity: HOME Plus, a randomized controlled trial. Int J Behav Nutr Phys Act. 2015;12:154–015–0320-3. 10.1186/s12966-015-0320-3 PMC4678662 26667110

[23] Fulkerson JA, Friend S, Horning M, Flattum C, Draxten M, Neumark-Sztainer D (2018). Family home food environment and nutrition-related parent and child personal and behavioral outcomes of the healthy home offerings via the mealtime environment (HOME) plus program: a randomized controlled trial. J Acad Nutr Diet.

[24] Sidebottom AC, Benson G, Vacquier M, Pereira R, Hayes J, Boersma P (2021). Population-level reach of cardiovascular disease prevention interventions in a rural community: findings from the heart of New Ulm project. Popul Health Manag.

[25] Sidebottom AC, Sillah A, Miedema MD, Vock DM, Pereira R, Benson G (2016). Changes in cardiovascular risk factors after 5 years of implementation of a population-based program to reduce cardiovascular disease: the heart of New Ulm project. Am Heart J.

[26] Flattum C, Friend S, Horning M, Lindberg R, Beaudette J, Fulkerson JA (2021). Family-focused obesity prevention program implementation in urban versus rural communities: a case study. BMC Public Health.

[27] Buscemi J, Kanwischer K, Becker AB, Ward DS, Fitzgibbon ML (2015). Society of Behavioral Medicine Health Policy C. Society of Behavioral Medicine position statement: early care and education (ECE) policies can impact obesity prevention among preschool-aged children. Transl Behavl Med.

[28] Fulkerson JA, Nelson MC, Lytle L, Moe S, Heitzler C, Pasch KE (2008). The validation of a home food inventory. Int J Behav Nutr Phys Act.

[29] Lohman T, Roche A, Martorell R (1988). Anthropometric standardization reference manual.

[30] Dietz WH, Gortmaker SL (2001). Preventing obesity in children and adolescents. Annu Rev Public Health.

[31] Ho M, Garnett SP, Baur L, Burrows T, Stewart L, Neve M (2012). Effectiveness of lifestyle interventions in child obesity: systematic review with meta-analysis. Pediatrics..

[32] Epstein LH, Paluch RA, Roemmich JN, Beecher MD (2007). Family-based obesity treatment, then and now: twenty-five years of pediatric obesity treatment. Health Psychol.

[33] Center for Disease Control: National Center for Health S. CDC growth charts, United States.

[34] USDA. Definitions of food security: United States Department of Agriculture; [Available from: https://www.ers.usda.gov/topics/food-nutrition-assistance/food-security-in-the-us/definitions-of-food-security/].

[35] Blumberg SJ, Bialostosky K, Hamilton WL, Briefel RR (1999). The effectiveness of a short form of the household food security scale. Am J Public Health.

[36] Flattum C, Draxten M, Horning M, Fulkerson JA, Neumark-Sztainer D, Garwick A, et al. HOME Plus: Program design and implementation of a family-focused, community-based intervention to promote the frequency and healthfulness of family meals, reduce children's sedentary behavior, and prevent obesity. Int J Behav Nutr Phys Act. 2015;12:53–015–0211-7. 10.1186/s12966-015-0211-7 PMC4417510 25925226

[37] Bandura A (1986). Social foundations of thought and action: a social cognitive theory.

[38] Elder JP, Lytle L, Sallis JF, Young DR, Steckler A, Simons-Morton D (2007). A description of the social-ecological framework used in the trial of activity for adolescent girls (TAAG). Health Edu Res.

[39] Klein EG, Lytle LA, Chen V (2008). Social ecological predictors of the transition to overweight in youth: results from the Teens eating for energy and nutrition at schools (TEENS) study. J Am Diet Assoc.

[40] Horning ML, Ostrow L, Beierwaltes P, Beaudette J, Schmitz K, Fulkerson JA (2020). Service learning within community-engaged research: facilitating nursing student learning outcomes. J Prof Nurs.

[41] Maniccia DM, Davison KK, Marshall SJ, Manganello JA, Dennison BA (2011). A meta-analysis of interventions that target children's screen time for reduction. Pediatrics..

[42] Waters E, de Silva-Sanigorski A, Hall BJ, Brown T, Campbell KJ, Gao Y, et al. Interventions for preventing obesity in children. Cochrane Database Syst Rev. 2011(12):Cd001871. 10.1002/14651858.CD001871.pub3 22161367

[43] Barlow SE (2007). Expert committee recommendations regarding the prevention, assessment, and treatment of child and adolescent overweight and obesity: summary report. Pediatrics..

[44] Durlak JA, DuPre EP (2008). Implementation matters: a review of research on the influence of implementation on program outcomes and the factors affecting implementation. Am J Comm Psychol.

[45] Bellg AJ, Borrelli B, Resnick B, Hecht J, Minicucci DS, Ory M (2004). Enhancing treatment fidelity in health behavior change studies: best practices and recommendations from the NIH behavior change consortium. Health Psychol.

[46] Wilfley DE, Saelens BE, Stein RI, Best JR, Kolko RP, Schechtman KB (2017). Dose, content, and mediators of family-based treatment for childhood obesity: a multisite randomized clinical trial. JAMA Pediatr.

[47] Daniels SR, Kelly AS (2014). Pediatric severe obesity: time to establish serious treatments for a serious disease. Child Obes.

[48] R: A language and environment for statistical computing Vienna, Austria: R Foundation for Statistical Computing; 2019 [Available from: https://www.R-project.org/.

[49] Miller WR, Rollnick S (2014). The effectiveness and ineffectiveness of complex behavioral interventions: impact of treatment fidelity. Contemp Clin Trials..

[50] Ells LJ, Rees K, Brown T, Mead E, Al-Khudairy L, Azevedo L (2018). Interventions for treating children and adolescents with overweight and obesity: an overview of Cochrane reviews. Int J Obes.

[51] Styne DM, Arslanian SA, Connor EL, Farooqi IS, Murad MH, Silverstein JH (2017). Pediatric obesity-assessment, treatment, and prevention: an Endocrine Society clinical practice guideline. J Clin Endocrinol Metab.

[52] Nobles J, Summerbell C, Brown T, Jago R, Moore T (2021). A secondary analysis of the childhood obesity prevention Cochrane review through a wider determinants of health lens: implications for research funders, researchers, policymakers and practitioners. Int J Behav Nutr Phys Act.

[53] Pedersen M, Brown B, Harris K, France S, Tryon M, Cooksley A. Rural Parent Support of Child Health Behavior in the Home Environment: A Qualitative Study on an American Indian Reservation. Glob Pediatr Health. 2019;6:2333794x19847451. 10.1177/2333794X19847451 PMC6506919 31106246

[54] O'Connor EA, Evans CV, Burda BU, Walsh ES, Eder M, Lozano P (2017). Screening for obesity and intervention for weight Management in Children and Adolescents: evidence report and systematic review for the US preventive services task force. JAMA..

[55] Brown T, Moore TH, Hooper L, Gao Y, Zayegh A, Ijaz S, et al. Interventions for preventing obesity in children. Cochrane Database Syst Rev. 2019;7(7):Cd001871. 10.1002/14651858.CD001871.pub4 PMC6646867 31332776

